# Dacron® Graft Kinking Following Ascending Aorta Replacement Is Not Only a Cosmetic Issue

**DOI:** 10.21470/1678-9741-2022-0468

**Published:** 2025-03-13

**Authors:** Ermal Likaj, Alfred Ibrahimi, Saimir Kuci, Jacob Zeitani

**Affiliations:** 1 Cardiac Surgery Department, Mother Teresa University, Tirana, Albania; 2 Anesthesiology and Reanimation Department, Mother Teresa University, Tirana, Albania; 3 Neurosciences and Rehabilitation Department, University of Ferrara, Ferrara, Italy

**Keywords:** Aortic Valve, Bicuspid Aortic Valve Disease, Polyethylene Terephthalates, Hemolysis, Surgical Anastomosis, Bacteria, Fatigue, Inflammation

## Abstract

A 58-year-old man, who has undergone ascending aorta replacement, started to complain of pain in the lower limbs, shortness of breath, and progressive fatigue a few months after surgery. Transthoracic and transesophageal Doppler echocardiographies revealed a diseased bicuspid aortic valve and a subocclusive mass in the ascending aorta. Thoracic computed tomography angiography confirmed the presence of a subocclusive mass, pseudoaneurysm formation, and a distorted shape of the Dacron^®^ graft. The patient underwent urgent surgery to remove the mass, which appeared to be a thrombus, and aortic valve and ascending aorta replacement. Kinking of vascular graft has been reported including surgical techniques to correct the excessive length to avoid gradients and guarantee laminar flow. When kinking is severe, high gradients and hemolysis can be detected. However, thrombus formation in the ascending aorta segment is less likely, due to the high blood velocity flow. Therefore, several concurrent causes should be considered. In this case, the most probable explanation for thrombus formation was kinking of a too long Dacron^®^ graft, combined with extrinsic compression effect of the graft by the pseudoaneurysm at the anastomosis site and anomalous flow directed from the diseased bicuspid aortic valve. Various grades of Dacron^®^ graft kinking might occur following ascending aorta replacement and undiagnosed at follow-up especially if resulting in mild symptoms, thus, careful visual and echocardiography evaluation should be done at the end of surgery. Finally, distorted Dacron^®^ graft might trigger thrombus formation when inflammation and coagulation processes are set off during bacteria or viral infection.

## CASE PRESENTATION

A 58-year-old man has undergone replacement of the ascending aorta in another hospital. A few months after surgery, the patient experienced pain in the lower limbs, shortness of breath, and progressive fatigue. Initially, due to coexistence of chronic type B aortic dissection, tests including repeated computed tomography angiography of the descending aorta and peripheral arterial echo Doppler were performed focusing on blood hypoperfusion of the lower limbs.

**Fig. 1:**
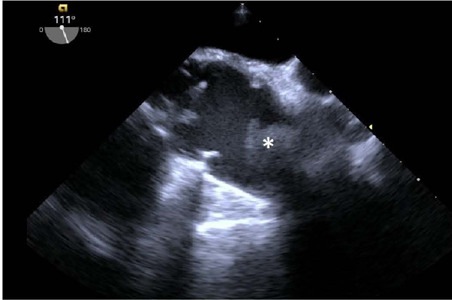
Transesophageal echocardiography showing the mass (*) in the ascending aorta.

**Fig. 2:**
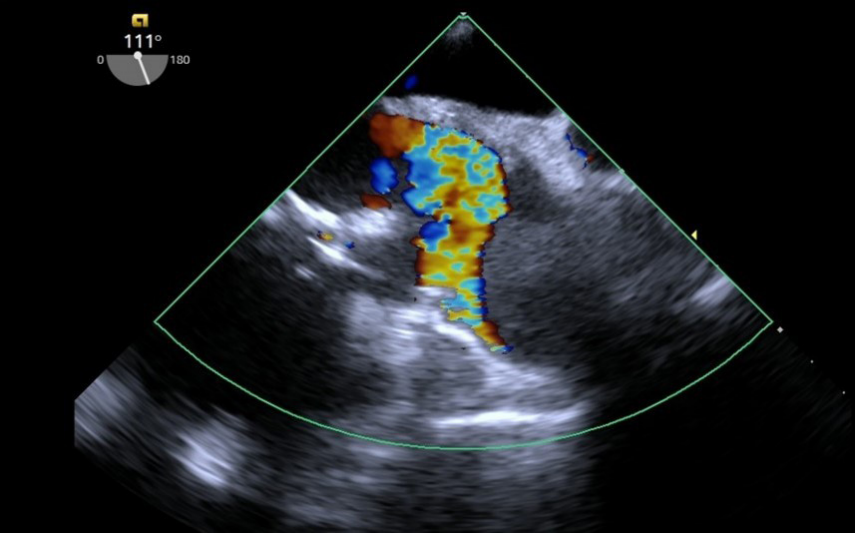
Transesophageal Doppler echocardiography showing the subocclusive ascending aorta lumen obstruction.

**Fig. 3:**
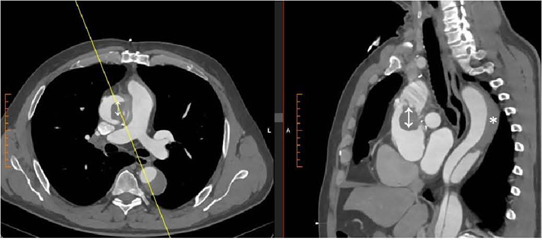
Computed tomography angiography showing mass formation (↨) in the ascending aorta and partial thrombosis of the false lumen (*).

**Fig. 4:**
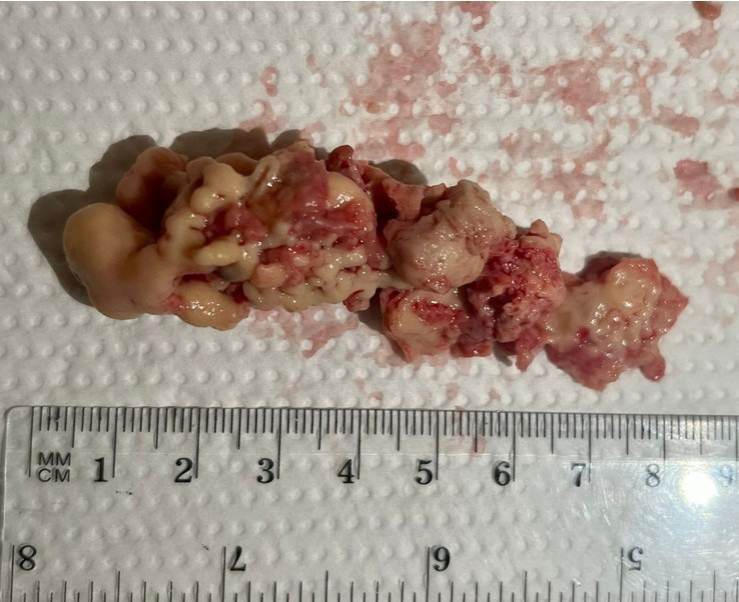
Material removed from the ascending aorta.

Six months after the first surgery, he was admitted to our hospital, where he underwent transthoracic and transesophageal two-dimensional Doppler echocardiography. This revealed a diseased bicuspid aortic valve (BAV) and a subocclusive mass in the ascending aorta (Figures 1 and 2 and Video 1). A thoracic computed tomography angiography confirmed the presence of a subocclusive mass which started at the level of the proximal anastomosis of the Dacron^®^ graft and extended up to the brachiocephalic trunk. Also, a pseudoaneurysm formation was detected, causing extrinsic compression on the vascular graft (Figure 3). The patient underwent urgent surgery to remove the mass, which appeared to be a thrombus, and aortic valve replacement (AVR) with mechanical prosthesis together with replacement of the residual distal native ascending aorta in circulatory arrest (Figure 4). The postoperative course was uneventful, and the patient reported improvement of his symptoms.

**Video 1:**
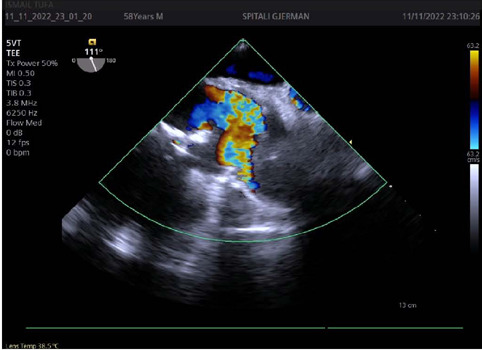
Transesophageal echocardiography showing accelerated flow because of mass obstruction in the ascending aorta.

*Link: https://youtu.be/LEXwxtrDEuw*

## DISCUSSION

Graft kinking following ascending aorta replacement is not infrequent and well documented, especially if only a short segment of the native aorta is replaced while the aorta is clamped and the heart is empty^[1]^. In order to resect all pathologic aortic wall, replacement of the ascending aorta with open distal anastomosis in circulatory arrest is mandatory. However, long cardiopulmonary bypass time, increased surgical risks, impaired hemostasis, and coagulation abnormalities should be considered^[2,3]^.

Unfavorable anatomy of the heart-aorta angulations might negatively affect the correct graft geometry. Also, diameter discrepancy between the proximal and distal aorta, common in Marfan syndrome patients, might lead to excessive graft length, resulting in significant kinking. The Dacron^®^ grafts are cylindrical and therefore are inevitably not fitting the small and large ascending aorta curvature morphology; thus, the graft should be trimmed obliquely, however still leaving some grade of discrepancy, which results in excessive fabric in the small curvature. Nevertheless, in worse cases, when severe kinking is seen or intraoperative transesophageal echocardiography (TEE) shows accelerated flow and gradients, graft length correction is needed.

The incidence of distorted graft might be underestimated and undiagnosed postoperatively due to overlooked intraoperative TEE, followed by none to mild symptoms. Indeed, this was commonly misdiagnosed prior to the patient’s arrival to our clinic. The physiopathology of thrombus formation in the large arterial vessels is not completely clear. In several case reports, thrombus formation is found in native aorta and attributed to atherosclerosis plaques and coagulation disorders.

Recently, coagulation disorders with thrombus formation in different vessels districts have been reported in coronavirus disease 2019 patients^[4]^. To our knowledge, this is the first report of a patient who underwent ascending aorta replacement and experienced thrombus formation in a very short time after surgery. The most probable explanation for the early formation of the huge thrombus was kinking of the too long Dacron^®^ graft combined with the effect of extrinsic pseudoaneurysm compression and the diseased BAV (Type 1b R-N), which led to an increasing blood turbulence.

Advances in cardiovascular magnetic resonance have suggested that the flow pattern in the ascending aorta in BAV causes an asymmetrical, off-center flow jet which hits the ascending aortic wall, leading to marked rotational helical flow. Altered flow patterns can be found in the ascending aorta of individuals with BAV compared with individuals with tricuspid aortic valve^[5,6]^. When open cardiac surgery procedures are performed, the treatment of non-severely diseased valves should be considered, balancing increased surgical risks. Verzini et al.^[7]^ evaluated the fate of untreated mild to moderate diseased BAV during ascending aorta replacement. According to their finding, up to 10 years after isolated ascending aorta replacement, the mild to moderate regurgitation/stenosis of a BAV did not do significantly worse, and therefore AVR is not mandatory. Eventually, in the present case, it was thought that it was more advisable not to replace the diseased aortic valve.

## CONCLUSION

Dacron® graft kinking following ascending aorta replacement, particularly in patients with BAV, might increase the risk of thrombus formation also in large vessels with high velocity flow. Therefore, intraoperative TEE should be performed also focusing on blood flow in the replaced Dacron^®^ graft, ensuring absence of a turbulent flow.

## Abbreviations, Acronyms & Symbols

AVR: Aortic valve replacement
BAV: Bicuspid aortic valve
TEE: Transesophageal echocardiography

## References

[1] Nezic D, Milicic M, Boricic M, Micovic S (2021). Modification of an old technique to correct kinking of tubular
graft interposed to reconstruct ascending aorta. Heart Lung Circ.

[2] Rathore K, Newman M (2022). Aortic root and distal arch management during type A aortic
dissection repair: expanding horizons. Braz J Cardiovasc Surg.

[3] Zeitani J, Buccisano F, Nardella S, Flaminio M, Prati P, Chiariello G (2013). Mini-extracorporeal circulation minimizes coagulation
abnormalities and ameliorates pulmonary outcome in coronary artery bypass
grafting surgery. Perfusion.

[4] Woehl B, Lawson B, Jambert L, Tousch J, Ghassani A, Hamade A (2020). 4 cases of aortic thrombosis in patients with
COVID-19. JACC Case Rep.

[5] Wiesemann S, Trauzeddel RF, Musa A, Hickstein R, Mayr T, von Knobelsdorff-Brenkenhoff F (2023). Changes of aortic hemodynamics after aortic valve replacement-A
four dimensional flow cardiovascular magnetic resonance follow up
study. Front Cardiovasc Med.

[6] Rodríguez-Palomares JF, Dux-Santoy L, Guala A, Kale R, Maldonado G, Teixidó-Turà G (2018). Aortic flow patterns and wall shear stress maps by 4D-flow
cardiovascular magnetic resonance in the assessment of aortic dilatation in
bicuspid aortic valve disease. J Cardiovasc Magn Reson.

[7] Verzini A, Bargagna M, Ascione G, Sala A, Carino D, Del Forno B (2021). Fate of mild-to-moderate bicuspid aortic valve disease untreated
during ascending aorta replacement. J Card Surg.

